# The Temporal Dynamics Model of Emotional Memory Processing: A Synthesis on the Neurobiological Basis of Stress-Induced Amnesia, Flashbulb and Traumatic Memories, and the Yerkes-Dodson Law

**DOI:** 10.1155/2007/60803

**Published:** 2007-03-28

**Authors:** David M. Diamond, Adam M. Campbell, Collin R. Park, Joshua Halonen, Phillip R. Zoladz

**Affiliations:** ^1^Medical Research Service, VA Hospital, Tampa, FL 33612, USA; ^2^Department of Psychology, University of South Florida, Tampa, FL 33620, USA; ^3^Department of Molecular Pharmacology and Physiology, University of South Florida, Tampa, FL 33612, USA

## Abstract

We have reviewed research on the effects of stress on LTP in the hippocampus, amygdala and prefrontal cortex (PFC) and present new findings which provide insight into how the attention and memory-related functions of these structures are influenced by strong emotionality. We have incorporated the stress-LTP findings into our “temporal dynamics” model, which provides a framework for understanding the neurobiological basis of flashbulb and traumatic memories, as well as stress-induced amnesia. An important feature of the model is the idea that endogenous mechanisms of plasticity in the hippocampus and amygdala are rapidly activated for a relatively short period of time by a strong emotional learning experience. Following this activational period, both structures undergo a state in which the induction of new plasticity is suppressed, which facilitates the memory consolidation process. We further propose that with the onset of strong emotionality, the hippocampus rapidly shifts from a “configural/cognitive map” mode to a “flashbulb memory” mode, which underlies the long-lasting, but fragmented, nature of traumatic memories. Finally, we have speculated on the significance of stress-LTP interactions in the context of the Yerkes-Dodson Law, a well-cited, but misunderstood, century-old principle which states that the relationship between arousal and behavioral performance can be linear or curvilinear, depending on the difficulty of the task.

## 1. INTRODUCTION

Numerous reviews in recent years have advanced our understanding of the interactions among long-term potentiation and depression (LTP/LTD), stress, and memory. These reviews have focused on specific topics, such as the cognitive implications of stress-LTP-LTD interactions (Kim and Diamond [1]; Diamond et al. [2]; Diamond et al. [3]; Kim et al. [4]), stress, LTP, and psychopathology (Post et al. [5]; McEwen and Magarinos [6]; Elzinga and Bremner [7]; Vermetten and Bremner [8]; Jay et al. [9]; Diamond et al. [10]; Buwalda et al. [11]), stress and metaplasticity (Abraham and Tate [12]; Kim and Yoon [13]), the effects of glucocorticoids on LTP (McEwen [14]; Garcia [15]; Joëls [16]), a comparison of stress effects on LTP in different brain regions (Diamond et al. [17]; Abe [18]; Richter-Levin and Akirav [19]; Richter-Levin [20]; Kim and Jung [21]; Akirav and Richter-Levin [22]), and a molecular analysis of stress-LTP interactions (Cremer et al. [23]; Popoli et al. [24]; Huang et al. [25]). Here, we have provided a different perspective on stress and LTP than has been considered previously. We have speculated on the functional significance of the finding that stress has different effects on LTP in different brain structures. Thus, stress has been shown to block the induction of LTP in the prefrontal cortex (PFC), and to enhance, as well as to impair, LTP in the hippocampus and amygdala. This review explores the idea that understanding the differential effects of stress on LTP in the PFC, hippocampus, and amygdala provides a framework towards understanding the neurobiological basis of flashbulb and traumatic memories, stress-induced amnesia, and the Yerkes-Dodson Law.

## 2. FLASHBULB MEMORIES AND VICISSITUDES OF THEWELL-CITED, BUT MISUNDERSTOOD, YERKES-DODSON LAW

One of the earliest researchers to comment on how emotion affects memory was James [26], who stated that “*an impression may be so exciting emotionally as almost to leave a scar upon the cerebral tissues*.” This early observation that strong emotionality can generate a long-lasting memory of the arousing event was also studied by Colgrove [27] in his descriptions of the recollections people had of emotionally charged events. Colgrove noted that most adults could describe, in great detail, events that had transpired on the day when they had learned, over three decades before, that President Lincoln had been assassinated. Other rapidly formed, vivid, and durable memories have been described by people who experienced events of great importance, such as assassinations of international leaders and the terrorist attacks on America on September 11, 2001 (Somer and Saadon [28]; Christianson [29]; Wright and Gaskell [30]; Terr et al. [31]; Kvavilashvili et al. [32]; van Giezen et al. [33]; Berntsen and Thomsen [34]; Curci and Luminet [35]). The powerful strengthening of memories of events occurring in times of strong emotionality was referred to as “hypermnesia” by Stratton [36] and then as “flashbulb memories” by Brown and Kulik [37].

**Figure 1:**
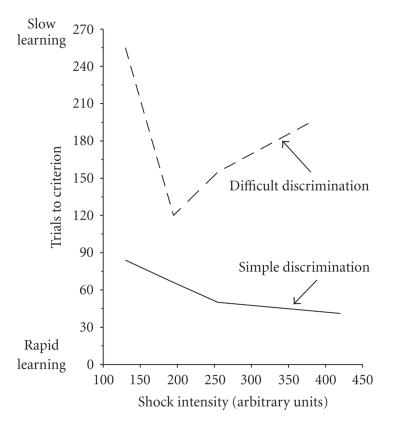
A subset of data from Yerkes and Dodson [38]. Mice were trained to avoid shock in a simple versus difficult visual discrimination task. The simple task involved a dark versus bright discrimination and the more difficult task involved a discrimination in which the two sides of the escape box were at similar levels of illumination. Behavioral performance increased linearly with increased levels of shock in the simple task, but performance was maximal at an intermediate level of shock for mice trained in the difficult discrimination.

A decade after Colgrove's description of the influence of emotion on memory, Yerkes and Dodson [38] studied the effects of different shock intensities on the rate of learning by mice in a discrimination avoidance task. These investigators showed that when mice were trained in a simple, that is, black/white, visual discrimination task to avoid shock, their rate of learning improved linearly with an increase in the intensity of the shock. When mice were trained in a more difficult, that is, black/gray, visual discrimination task, their rate of learning was more efficient with an intermediate intensity of shock than with the highest intensity of shock. Their findings, which were then replicated separately by Yerkes [39] and later by Dodson [40], became known as the Yerkes-Dodson Law, which essentially stated that a high level of motivation can enhance learning on an easy task and impair learning on a difficult task (see also Yerkes [39]). Figure 1 provides a subset of the data from the Yerkes and Dodson [38] study, which illustrates the finding that the relationship between shock intensity and performance on the task was linear (increased shock intensity produced increased performance) for the simple discrimination and nonlinear (an intermediate intensity of shock produced optimal performance) for the complex discrimination.

With rare exceptions (Ni [41]; Young [42]; Postman [43]), the work of Yerkes and Dodson and the law it spawned were largely ignored in the first half of the twentieth century. Five decades passed from the formation of the Yerkes-Dodson law before it was first tested by Broadhurst [44] with modern techniques and statistical data analyses. In Broadhurst's work, rats were trained to escape from submersion in water in a task with different levels of difficulty and motivation. Broadhurst showed that rats tested on an easy visual discrimination task learned rapidly when they were trained with the highest level of motivation (stress). He also showed that an intermediate degree of stress produced the best performance in rats trained on a more difficult version of the task. Thus, Yerkes and Dodson [38] and then Broadhurst [44] demonstrated that high levels of stress impaired performance on a difficult, but not on an easy, task. Other studies on people and rodents have reinforced the notion of the importance of taking into account the difficulty of the task as an intervening variable in arousal effects on performance (e.g., Dickman [45]; Hammes [46]; Denenberg and Karas [47]; Telegdy and Cohen [48]; Bregman and McAllister [49]; Anderson [50]; Mesches et al. [51]; Diamond et al. [52]).

In the 1950s, major figures in the field of cognitive psychology appear to have been unaware of, or ignored, the findings of Yerkes and Dodson when they stated that the relationship between arousal and performance was exclusively curvilinear. Thus, Schlosberg [56], Hebb [53], and Duffy [57] all asserted, without reference to Yerkes and Dodson, that there is a curvilinear relationship between arousal and performance. For example, Hebb's [53] view was that “there seems no doubt: the *(right side of the arousal-performance curve)* must come down to a low level” (page 251). Similarly, Duffy [57] stated that “the optimal degree of activation appears to be a moderate one, the curve which expresses the relationship between activation and quality of performance taking the form of an inverted U” (page 268).

The exclusion of the findings of Yerkes and Dodson in these reviews cannot be explained by a complete loss of interest in the Yerkes-Dodson law by the mid-twentieth century. At about this time, Postman [43] provided an exhaustive review of animal and human research conducted in the first half of the twentieth century on emotion and learning. He cited the findings of Yerkes and Dodson when he stated that “relatively severe punishment (intensive shock) is most effective in learning simple habits such as black-white discrimination … and relatively mild punishment is optimal in the case of difficult tasks, such as complex types of discrimination” (page 507). Similarly, Harlow ([58, page 27]) noted, in his application of the Yerkes Dodson law to primate learning, that the “intensity of nociceptive stimulation may be positively related to the speed of formation of conditioned avoidance responses … but the use of intense nociceptive stimulation prevents the monkey from solving any problem of moderate complexity.” Thus, the idea that arousal interacts with task difficulty to positively or negatively influence performance was well established in cognitive psychology in the first half of the twentieth century.

**Figure 2:**
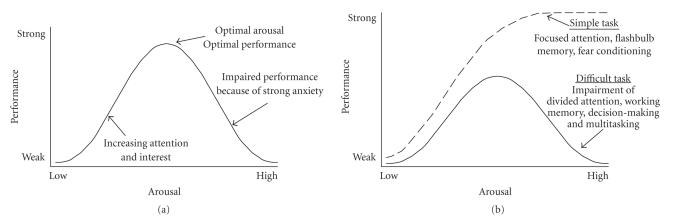
A comparison of the Hebbian version of the Yerkes-Dodson law, as it has been commonly represented for the past 50 years (a), and the original version, based on the actual findings and theorizing of Yerkes and Dodson ([38]; (b)). The Hebbian version incorrectly states that high levels of stress, anxiety, or motivation produce a monolithic impairment of performance. The original version based on the actual [38] Yerkes-Dodson findings takes into account the finding that strong emotionality can enhance performance under “simple” learning conditions, such as when learning involves focused attention on a restricted range of cues, and impairs performance under more complex or challenging learning situations, such as in divided attention, multitasking, and working memory tasks. Graph (a) is adapted from 5 decades of publications and books, for example, Hebb [53], Loftus [54], and Radvansky [55].

It is ironic that not only were the original findings of Yerkes and Dodson ignored in major reviews on emotion and learning in the 1950s, but Hebb's incomplete illustration of the arousal-performance relationship as exclusively curvilinear (Figure 2 in Hebb [53]) incorrectly came to be known as the Yerkes-Dodson law by later researchers. Beginning in the 1960s (e.g., Broadbent [59]), the Yerkes-Dodson law devolved into a single inverted U-shaped curve, which has been promulgated, to this day, by introductory psychology textbooks (e.g., Radvansky [55]). Even contemporary scholars in the field of emotion, brain, and memory have relegated the linear component of the original Yerkes-Dodson law to the status of a mere footnote (Christianson [29]) or they have disregarded it entirely, focusing solely on the Hebbian view that a single inverted-U shaped curve represents how arousal interacts with cognition (e.g., Loftus [54]; Neiss [60]; Metcalfe and Jacobs [61]; Aston-Jones et al. [62]; Mendl [63]; Aston-Jones et al. [64]; Morris [65]).

As one of us noted recently (Diamond [66]), debates have raged for the past 5 decades regarding the validity of the Yerkes-Dodson law, but it is primarily the incomplete (Hebbian) version of the Yerkes-Dodson law that has become one of the most debated and even vilified doctrines in cognitive psychology (Deffenbacher [67]; Neiss [60]; Christianson [29]; Baumler [68]; Teigen [69]; Watters et al. [70]; Dutton and Carroll [71]; Hanoch and Vitouch [72]). Thus, the Yerkes-Dodson law suffered the indignity to be largely ignored during the first half of the twentieth century, and once it was revived, to be misrepresented to the present day. This five-decade-long misrepresentation of Yerkes and Dodson's findings has occurred despite the unambiguous statement by these authors that “an easily acquired habit may be readily formed under strong stimulation, whereas a difficult habit may be acquired only under relatively weak stimulation. That this fact is of great importance to students of animal behavior and animal psychology is obvious” (Yerkes and Dodson [38, pages 481-482]). With its thousands of reference citations in the past century, Yerkes and Dodson [38] may have the dubious distinction to be the most highly cited, but largely unread, paper in the history of science.

In a rare example of a scholarly analysis of the Yerkes-Dodson law, Hanoch and Vitouch [72] assessed a half century of misdirection by stating that “what Yerkes and Dodson had in mind was more sophistication than what their U-entranced successors made of it … later generations let the law collapse into one single curve with its idealized and highly abstract, quasiunidimensional axes” (see also Teigen [69, pages 430-431] for related discussion). As we approach the 100th anniversary of the publication of their study, we honor Yerkes and Dodson with a representation of a subset of the data from their [38] paper in Figure 1, which illustrates the linear and curvilinear (task difficulty-dependent) aspects of their findings. In addition, we have provided our version of the original (dual linear/curvilinear) and near-ubiquitous, Hebbian (curvilinear), version of the Yerkes-Dodson law in Figure 2.

Whereas leaders in the field of cognitive psychology have fiercely debated the heuristic value of the Hebbian version of the Yerkes-Dodson law, behavioral neuroscientists, by contrast, have universally accepted and incorporated the Hebbian version of the Yerkes-Dodson law into their theorizing on brain-emotion interactions (e.g., Foy et al. [73]; Diamond et al. [74]; LeDoux [75]; Metcalfe and Jacobs [61]; Aston-Jones et al. [62]; Mendl [63]; Aston-Jones et al. [64]; Richter-Levin [20]; Elzinga et al. [76]; Andreano and Cahill [77]; Morris [65], but see Schulteis and Martinez [78]). A recent study provides an example of the application of the Hebbian version of the Yerkes-Dodson law to behavioral neuroscience research. Andreano and Cahill [77] found an inverted-U-shaped relationship between cortisol levels and memory consolidation in people, that is, an intermediate level of cortisol correlated with peak memory performance. These investigators stated that their findings were consistent with the Yerkes-Dodson law, which, according to them, would predict that there should be a curvilinear relationship between cortisol levels and memory performance (pages 467–469). Actually, the Yerkes-Dodson law does not make this prediction. The Yerkes-Dodson law, in its original form, would predict that on simple tasks, stress levels of cortisol should enhance memory, and on more complex tasks, stress levels of cortisol should impair memory. Consequently, Andreano and Cahill's findings are relevant, specifically, towards enhancing our understanding of the stress side of the curvilinear component of the Yerkes-Dodson law. A more thorough understanding of how cortisol interacts with memory would need to address how high levels of cortisol (or corticosterone, the rodent form of cortisol) and drugs that activate cortisol receptors interact with task difficulty to impair, as well as enhance, memory consolidation (Sandi et al. [79]; Sandi [80]; Cordero and Sandi [81]; Buchanan and Lovallo [82]; Cordero et al. [83]; Rimmele et al. [84]; Hui et al. [85]; Het et al. [86]).

We introduced this section by mentioning “flashbulb memories,” which are durable memories formed in response to strong emotional experiences. Had Schlosberg, Duffy, and Hebb been correct in their assertion that strong emotion reliably impairs cognition, then flashbulb memories should not exist. That is, if the right (high stress) side of the stress-performance curve always declines to produce poor performance, as it does in the Hebbian version of the Yerkes-Dodson law (Figure 2(a)), then strong emotionality should universally impair all forms of cognition. On the contrary, extensive research has shown that strong emotionality can, under some conditions, enhance memory (Ni [41]; Cahill et al. [87]; McGaugh [88]; Sharot et al. [89]; Nielson et al. [90]). The well-described flashbulb memory phenomenon is just one example of how arousing experiences can strengthen memories. Although emotional memories may not be flawless representations of the original experiences (Tekcan et al. [91]; Schmidt [92]; Laney and Loftus [93]; Loftus [94]), their general accuracy and durability which can span decades are remarkable (Tekcan and Peynircioğlu [95]; Berntsen and Thomsen [34]).

In summary, we have asserted that the Hebbian version of the Yerkes-Dodson law (Figure 2(a)) is an incomplete representation of the effects of emotionality on cognition because it does not address how memories can be strengthened by strong emotional experiences. Traumatic experiences place a subject at the highest right side of the arousal-performance curves depicted in Figure 2, and they can generate intrusive memories so powerful and durable that they can have long-lasting pathological consequences which underlie anxiety and mood disorders, including depression and post-traumatic stress disorder (PTSD) (Ehlers and Clark [96]; Layton and Krikorian [97]; Rubin et al. [98]; Ehlers et al. [99]; Bremner [100]; Michael et al. [101]; Nemeroff et al. [102]). Only the original version of the Yerkes-Dodson law (Figure 2(b)) can incorporate the finding that emotional trauma can produce an enhancement of memory. Hence, the original version of the Yerkes-Dodson law is of greater value to behavioral and psychiatric research than the Hebbian version because it incorporates the enhancement, as well as impairment, of memory in times of strong emotionality.

## 3. EASTERBROOK'S “CUE UTILIZATION” HYPOTHESIS: A CUE-BASED DISTINCTION BETWEEN SIMPLE AND COMPLEX TASKS

A problem with “task difficulty” as a critical factor in understanding emotion-memory interactions is that it is a subjective measure. It is therefore difficult, if not impossible, to operationally define the term “task difficulty” with objective criteria. Nevertheless, investigators over the past 5 decades have taken on this task. One of the earliest attempts to understand how task variables interact with performance was accomplished in a landmark paper by Easterbrook [103], in one of the most comprehensive and insightful analyses of how emotion affects cognition. Easterbrook assessed the influence of emotionality on cue utilization and the organization of behavior. He noted that strong emotionality “acts consistently to reduce the range of cues that an organism uses, and that the reduction in the range of cue utilization influences action in ways that are either organizing or disorganizing” (page 183). “On some tasks, reduction in the range of cue utilization *under high stress conditions* improves performance. *In these tasks*, irrelevant cues are excluded and *strong emotionality* is motivating. In other tasks, proficiency demands the use of a wider range of cues, and *strong emotionality* is disorganizing. There seems to be an optimal range of cue utilization for each task” (pages 197-198). Importantly, Easterbrook interpreted these observations as an indication that “the effect *of emotionality* on proficiency would depend on the complexity of the *task* studied” (page 187). Easterbrook emphasized that performance on only the most demanding and complex tasks would suffer a “disintegration” *(i.e., severe impairment)* as a result of strong emotionality (page 187; text in italics are paraphrased). He noted that there was an impairment in behavioral performance in complex tasks in response to strong emotionality because “the range of cue utilization is reduced in response to strong emotion” (page 197), and that “tasks requiring the use of smaller numbers of cues were facilitated by drive increments” (page 192).

Easterbrook's cue utilization hypothesis stated that with increased emotionality, there is a reduction in the range of cues that an individual can process. According to Easterbrook, if a task is complex, that is, involves attention to multiple cues, then performance will deteriorate under conditions of high stress. If, on the other hand, a task is simple, that is, involves focused attention to a single cue, as occurs, for example, with the “weapon focus” phenomenon (Christianson [29]; Safer et al. [104]; Pickel [105]), then performance will improve under high-stress conditions.

Easterbrook's approach towards identifying systematic relationships between cue utilization and emotionality has been fruitful in understanding how emotionality affects behavioral performance in people and rodents (Telegdy and Cohen [48]; Geen [106]; Cohen et al. [107]; Christianson [29]; Hanoch and Vitouch [72]). Thus, Easterbrook's cue utilization hypothesis and the original version of the Yerkes-Dodson law are complementary explanations for the finding that strong emotionality can enhance performance on a simple task and impair performance on a complex task.

We will return to the challenge of how to distinguish simple versus complex levels of task difficulty and how they relate to performance in a later section addressing the cognitive functions of the prefrontal cortex. First, we will review literature on the effects of stress on synaptic plasticity in different forebrain structures, and then we will present a physiological model which may prove to be of value in explaining how stress can impair memory and can also generate flashbulb memories.

## 4. EFFECTS OF STRESSON LTP IN THE HIPPOCAMPUS, AMYGDALA, AND PREFRONTAL CORTEX

Most of the research on stress and LTP has focused on the CA1 and dentate gyrus regions of the hippocampus, with a lesser volume of work on the PFC and amygdala. In addition, most of the stress-LTP studies have been conducted on male rats. This is an important issue because female rats and women appear to respond differently to acute stress than do the males of each species, a finding which was first suggested by Stratton [36] and then substantiated in contemporary research (Shors [108]; McEwen [109]; Beiko et al. [110]; Conrad et al. [111]; Kudo et al. [112]; Shansky et al. [113]; Cahill [114]). Therefore, we acknowledge that our speculation here is based primarily on research conducted on the hippocampus of male rats. The extension of this synthesis to females, in general, and to amygdala and PFC processing, in particular, needs to be substantiated with additional research.

Another issue worth mentioning is the potential role of long-term depression (LTD) in stress and memory processing. Elsewhere, Diamond et al. [2, 3] and others (Xu et al. [115]; Abraham and Tate [12]; Kim and Yoon [13]; Braunewell and Manahan-Vaughan [116]; Kemp and Manahan-Vaughan [117]; Sajikumar and Frey [118]; Huang et al. [25]) have speculated on the potential significance of stress-LTD interactions in hippocampal functioning. However, as the hypothesis we have presented here is at an early stage of development, we have restricted our speculation to the potential link between LTP and emotional memory processing.

Finally, we have arrived at the critical question that underlies the basis of our theorizing: what does it mean, from physiological and cognitive perspectives, for stress to affect the induction of LTP? Our approach to addressing this question is different from the conventional view that LTP can be understood exclusively as a physiological model of memory. We suggest here, as in previous theorizing (Diamond et al. [17]), that the successful versus unsuccessful induction of LTP can serve as a “diagnostic” measure with which to assess the functional state of a brain structure. If, for example, the induction of hippocampal LTP is enhanced 2 minutes after a rat is placed in a novel environment, then we would interpret this finding as evidence that hippocampal information processing has been enhanced by novelty, but the interpretation applies only to the influence of novelty on the hippocampus at the 2-minute time point. If, on the other hand, the induction of hippocampal LTP is blocked 30 minutes after a rat is placed in a novel environment, then we would interpret that finding narrowly, as well, as evidence that hippocampal information processing is inhibited 30 minutes after exposure to novelty. In this example, exposure of a rat to a novel environment, per se, does not generate a global excitatory or inhibitory effect on hippocampal functioning. Rather, it produces both effects, with each effect occurring at different times after the onset of the arousing experience. Therefore, the successful versus unsuccessful induction of LTP can serve as a diagnostic test to reveal whether the hippocampus has been *transiently* shifted into an enhanced or impaired state of plasticity induction at different times after the onset of an emotional experience.

With this diagnostic perspective on LTP induction in mind, we can now address the functional significance of the finding that stress blocks the induction of hippocampal LTP. In 1990, our group suggested that the reason why stress blocks LTP was because stress, itself, activates endogenous mechanisms of plasticity in common with mechanisms that are also activated by exogenously induced LTP (Diamond et al. [119]). We hypothesized that the stress-induced saturation of endogenous mechanisms of plasticity would render plasticity mechanisms refractory in response to subsequent stimulation. The stress-induced activation, followed by inhibition, of hippocampal plasticity mechanisms would thereby explain why stress interferes with the induction of LTP. Our hypothesis was supported by later work which revealed commonalities between the mechanisms underlying stress and tetanizing (LTP-inducing) effects on plasticity (discussed further by Shors and Dryver [120]; Diamond et al. [2]; Diamond et al. [3]; Huang et al. [25]). According to this view, stress blocks the induction of LTP because the tetanizing stimulation was delivered when the hippocampus was in a refractory phase for plasticity induction, which occurs following an initial stress-induced activation of NMDA-receptors. Support for this hypothesis is the finding that NMDA receptor blockade during stress blocks the stress-induced suppression of LTP (Kim et al. [121]).

In the following sections, we have extended our earlier speculation that stress activated endogenous mechanisms in common with LTP in the hippocampus with the hypothesis that the hippocampus and amygdala both undergo a stress-induced activation, followed by an inhibition, of mechanisms underlying synaptic plasticity. We suggest that the rapid activation of plasticity mechanisms in these two structures underlies the well-described, arousal-induced enhancement of memory, producing flashbulb and traumatic memories in people, and fear conditioning in rodents. We also hypothesize that the PFC does not undergo a stress-induced enhancement phase followed by an inhibitory phase. We interpret the stress-induced inhibition of LTP in the PFC as an indication that stress produces an immediate inhibition of the functioning of the PFC, which is revealed behaviorally as a narrowing of attention and impaired multitasking, or more globally, as an impairment of complex learning.

## 5. STRESS BLOCKS HIPPOCAMPAL LTP, THEREFORE STRESS IMPAIRS HIPPOCAMPAL FUNCTIONING

For almost four decades, Bruce McEwen's group has been studying how stress hormones affect the brain and behavior. He and his coworkers first showed that the hippocampus has the greatest density of glucocorticoid receptors of all brain structures (McEwen et al. [122]; McEwen et al. [123]), indicating that the hippocampus was highly responsive to stressful experiences. Subsequent glucocorticoid-related behavioral work from his group led to the conclusion that “hippocampal function may indeed be suppressed during periods of prolonged stress” (Micco et al. [124, page 328]). This view of stress interfering with hippocampal functioning was incorporated into theorizing by Jacobs and Nadel [125] as an explanation of how stress reactivates childhood fears and phobias. These authors speculated that phobias can develop during infancy, before the hippocampal “locale” system, with its context-specific learning system, develops. They suggested that “under severe stress, behavioral control devolves on the taxon *(nonhippocampal)* systems that are, in this state, unusually sensitive …” (page 518, text in italics added). They further proposed that “stress disrupts the function of the hippocampally based locale system and its context-specific learning capacities while potentiating taxon systems and their context-free associations” (page 518), and that the stress-induced suppression of the hippocampus would enable phobias that had been formed in childhood to be expressed in adulthood.

The first electrophysiological evidence that stress inhibited hippocampal functioning was provided by Richard Thompson and his coworkers, with their finding that stress (restraint with or without tail shock) blocked the induction of LTP in CA1 in vitro (Foy et al. [73]). They interpreted their findings of a stress-induced blockade of hippocampal LTP within the context of the Hebbian version of the Yerkes-Dodson law by stating that “cognitive performance deteriorates at extreme levels of arousal (which was) recognized by Yerkes and Dodson” (page 145). Their discussion provided the first suggestion that the stress-induced suppression of LTP could be linked to the presumed stress-induced impairment of hippocampal functioning.

At about the time that Thompson's group was studying restraint stress and paw shock effects on LTP in vitro, our group was investigating how stress affected a low threshold form of LTP in CA1 of behaving rats. This form of plasticity, which is referred to as primed burst (PB) potentiation, can be induced by a total of only 5 physiologically patterned pulses delivered to CA1 (Rose and Dunwiddie [126]; Diamond et al. [127]; and see also Larson and Lynch [128]; Larson et al. [129]; Staubli and Lynch [130] for related work). We found that the induction of PB potentiation was blocked in rats that were exposed to an unfamiliar environment (Diamond et al. [119]; Diamond et al. [131]). We also showed that when rats were explicitly acclimated to the environment, as indicated by a significant reduction in their levels of serum corticosterone, the blockade of PB potentiation was no longer present (Diamond et al. [131]). Importantly, when these same rats were then exposed to a second, stress-provoking (corticosterone-elevating) environment, once again, PB potentiation was suppressed. These findings demonstrated that the capacity for the hippocampus to generate plasticity, and presumably its memory storage functioning, was continuously influenced by an animal's emotional state.

Thus, the nascent stress-LTP field in the 1980s and early 1990s, led by McEwen's early research on hippocampal sensitivity to glucocorticoids (in conjunction with his pioneering work with Robert Sapolsky on the stress- and glucocorticioid-induced increases in the susceptibility of the hippocampus to damage; Sapolsky et al. [132]), the electrophysiological studies on the stress-induced suppression of LTP and PB potentiation (reviewed in Diamond and Rose [133]), and the theorizing by Jacobs and Nadel [125] on the psychopathological effects of stress on the hippocampus, all fully supported the view that stress exerts a disruptive influence on hippocampal functioning.

The hypothesis that stress inhibited hippocampal functioning was supported by a large number of cognitive and electrophysiological studies conducted in the past decade. For example, we have found that stress, involving exposure of rats to either an unfamiliar environment or to a predator, impaired hippocampus-dependent memory (Diamond et al. [134]; Diamond et al. [52]; Woodson et al. [135]; Sandi et al. [136]; Diamond et al. [137]; Park et al. [138]) and blocked the induction of PB potentiation in vivo (Diamond et al. [139]; Vouimba et al. [140]) and in vitro (Mesches et al. [51]). Our findings are consistent with recent work from other laboratories indicating that acute stress or corticosterone administration blocks hippocampal LTP (Shors et al. [141]; Shors and Thompson [142]; Pavlides et al. [143]; Pavlides et al. [144]; Pavlides et al. [145]; Garcia et al. [146]; Pavlides and McEwen [147]; Akirav and Richter-Levin [148]; Zhou et al. [149]; Wang et al. [150]; Garcia [15]; Kim et al. [151]; Alfarez et al. [152]; Xiong et al. [153]; Jay et al. [9]; Kim et al. [154]; Krugers et al. [155]; Wiegert et al. [156]) and can impair hippocampus-specific memory processing in rats (de Quervain et al. [157]; Conrad et al. [158]; Roozendaal et al. [159]) and people (Kirschbaum et al. [160]; de Quervain et al. [161]; Wolf et al. [162]; Payne et al. [163]; Buss et al. [164]; Wolf et al. [165]; Elzinga et al. [76]; Kuhlmann et al. [166]; Kuhlmann et al. [167]; Payne et al. [168]; Buchanan et al. [169]).

An illustration of the widespread acceptance of the idea that strong stress impairs hippocampal functioning was in statements by LeDoux [75] in his scholarly and widely read book on the brain and emotion. He commented that memory “may be interfered with if stress is sufficiently intense and prolonged to raise the level of adrenal steroids to the point where the hippocampus is adversely affected,” and he further suggested that “if the hippocampus was completely shut down by the stress to the point where it had no capacity to form a memory during the event, then it will be impossible through any means to dredge up a conscious memory of the event” (pages 243-244). Similar views of how traumatic experiences affect the hippocampus were expressed by van der Kolk [170], who suggested that “extreme emotional arousal interferes with hippocampal memory functions” (page 282), and by Joseph [171, 172] who stated that “under conditions of overwhelming terror, the hippocampus becomes desynchronized … what is experienced may be forgotten or stored abnormally and independently of the hippocampus … emotional memory and recall are in part mediated by the amygdala” ([171, page 175]).

The pervasive view in the 1990s that stress impairs hippocampal functioning and enhances amygdala functioning led Metcalfe and Jacobs [61] to propose a novel hypothesis which addressed the neurobiological basis of traumatic memory formation. These investigators categorized brain memory systems in terms of whether brain structures were activated (hot) or impaired (cool) by strong emotionality. According to Metcalfe and Jacobs [61], the amygdala is a component of the “hot” memory system, because it functions optimally under emotionally intense conditions. The hippocampus, by contrast, is a component of the “cool” memory system because it functions optimally under emotionally neutral conditions and is impaired by traumatic stress. The theorizing by Metcalfe and Jacobs [61], as well as by Nadel and Jacobs [173], were consistent with LeDoux's [75] speculation that stress induces a “shutdown of the hippocampus” (page 246), and “may even enhance amygdala functions” (page 245).

Metcalfe and Jacobs [61] also noted that intermediate levels of stress appeared to have a facilitatory effect on hippocampal plasticity. This view was based, in part, on the finding of an inverted-U-shaped relationship between the level of serum corticosterone and the magnitude of hippocampal PB potentiation or LTP (Bennett et al. [174]; Diamond et al. [74]; Kerr et al. [175]). That is, the magnitude of hippocampal synaptic plasticity was maximal in animals with intermediate levels of corticosterone, and was the lowest in animals with either low or high (stress) levels of corticosterone. In addressing the significance of this finding, Diamond et al. [74] and Metcalfe and Jacobs [61] perpetuated the misrepresentation of the Yerkes-Dodson law by suggesting that the U-shaped relationship between PB potentiation and corticosterone was a physiological manifestation of the (Hebbian version of the) Yerkes-Dodson law (Figure 2(a)).

This overview of studies on stress and hippocampal plasticity summarizes the view of many researchers over the past two decades that strong stress inhibits hippocampal functioning (e.g., Jacobs and Nadel [125]; van der Kolk [176]; Diamond and Rose [133]; LeDoux [75]; van der Kolk [170]; Nadel and Jacobs [173]; Kim and Yoon [13]; Joseph [172]; Diamond and Park [177]; Garcia [15]; Layton and Krikorian [97]; Kim and Diamond [1]; Lynch [178]; Diamond et al. [2]; Diamond et al. [3]; Kim and Jung [21]; Akirav and Richter-Levin [22]). In the next section, we will present a new perspective on this issue by integrating a broader range of research on stress-hippocampus-LTP interactions than has been considered previously.

## 6. CRACKS IN THE EDIFICE OF THE HYPOTHESIS THAT STRONG EMOTIONALITY GLOBALLY SUPPRESSES HIPPOCAMPAL FUNCTIONING

As discussed above, research conducted over the past two decades has demonstrated conclusively that stress blocks the induction of hippocampal synaptic plasticity (LTP and PB potentiation) and impairs spatial and declarative memory. Based on these findings, major figures in the field have stated that stress adversely affects hippocampal functioning. For example, according to Nadel and Jacobs [173], “high levels of stress impair the functioning of the hippocampus, weakening or totally disrupting those aspects of spatial and explicit memory subserved by this structure. A number of studies, with both humans and animals, have demonstrated this now well-accepted fact” (page 155). This perspective was discussed further by Metcalfe and Jacobs [61], who stated that memory processing was accomplished by the amygdala, and not by the hippocampus, during times of stress. These authors speculated that during traumatic stress, the hippocampus “becomes dysfunctional” (page 205). Similarly, Diamond et al. [17] and Layton and Krikorian [97] hypothesized that the amygdala becomes activated and temporarily stores information as the hippocampus is rendered nonfunctional during a traumatic experience. More recently, Akirav and Richter-Levin [22] summarized the consensus viewpoint by stating that “under certain stressful conditions, emotional memory storage in the amygdala will be facilitated at the expense of hippocampus-dependent spatiotemporal processing” (page 29).

Finally, perhaps the ultimate denial of a necessary role of the hippocampus in emotional memory processing was stated by Dalgleish [179], in his review of the history of research on affective neuroscience. Dalgleish discussed MacLean's [180] introduction of the term “limbic system,” which is still currently in use to describe the group of brain structures considered to be involved in emotion (but see commentary by LeDoux [75]). According to MacLean, the hippocampus was the core structure of the limbic system, responsible for integrating visceral with external information. Dalgleish, however, justified the expulsion of the hippocampus from the limbic system because it had only a relatively small role in emotionality, as it was “more involved in higher cognitive processes” (page 584).

We now suggest that the idea that hippocampal functioning is globally impaired by strong emotionality is incomplete and inaccurate. The following observations illustrate inconsistencies with the idea that strong stress impairs hippocampal functioning.

1. The hippocampus is an important component of contextual fear conditioning (Phillips and LeDoux [181]; Maren [182]; Sanders et al. [183]; Rudy et al. [184]). Moreover, hippocampal cells exhibit plasticity of their place fields in response to contextual fear conditioning (Moita et al. [185]; Moita et al. [186]), leading these authors to conclude that hippocampal “place cell remapping was related to the rat's learned fear of the environment” (Moita et al. [186, page 7015]). Fear conditioning training has stress-provoking elements which have been shown to block LTP and PB potentiation, such as exposure of rats to a novel environment (the training context) and electric shock, and yet, the formation of the contextual component of the fear memory is dependent on the integrity of the hippocampus. How is it possible for the hippocampus to exhibit fear-induced place cell plasticity and to form a contextual memory of a fear-provoking experience when fear suppresses hippocampal functioning?
2. Researchers outside of the stress-LTP field have long contended that activation of the amygdala exerts a facilitating effect on memory-related processing by other brain regions, including the hippocampus (McGaugh et al. [187]; Roozendaal et al. [188]; Nathan et al. [189]). In one example, Packard and Teather [190] demonstrated that the amphetamine-induced activation of the amygdala enhanced hippocampus-dependent spatial memory. In related work, neuroimaging studies have provided strong support for the idea that the conjoint activation of the hippocampus and amygdala under arousing counditions is a critical component of emotional memory storage and retrieval processes (Maratos et al. [191]; Dolcos et al. [192]; Dolcos et al. [193]). The finding that activation of both the amygdala and hippocampus is necessary for the formation of an emotional memory is incompatible with the view that stress “shuts down” the hippocampus.
3. Flashbulb memories are highly durable, explicit recollections of the details of events that had transpired during emotional experiences (Brown and Kulik [37]; Schmidt [92]). A traumatic memory is a type of flashbulb memory which is generated in response to a horrific and possibly life-threatening event. According to van der Kolk [170, 176], the suppression of hippocampal functioning and activation of the amygdala during horrific experiences underly the implicit, fragmented, and primarily sensory structure of traumatic memories. Traumatic memories certainly have a powerful implicit (nondeclarative) component, and PTSD patients commonly have amnesia, or “memory gaps,” for events that occurred during their trauma (van der Kolk et al. [194]; van der Kolk [176]; van der Kolk [170]; Joseph [171]; Yovell et al. [195]; Michael et al. [196]; Ehlers et al. [197]). However, traumatized people commonly provide explicit (declarative) descriptions of the event(s) that precipitated their PTSD symptoms. For example, Ehlers et al. [198] noted that PTSD patients could describe sensory elements of their traumatic experiences, such as a victim of a motor vehicle accident described hearing the sound of crunching metal which occurred during the accident, and a rape victim described the feel of the rapist's hands over her eyes. The ability of PTSD patients to verbally describe features, albeit only fragments, of their traumatic experiences suggests that their memories of trauma are not entirely implicitly based. If hippocampal functioning actually was shut down during emotional experiences, then emotional memories would be similar to those observed in amnesics with temporal lobe damage. That is, an individual with a complete loss of the hippocampal functioning, such as HM, can acquire implicit information, such as perceptual and motor skills, but completely lacks an explicit memory of the learning experience (Scoville and Milner [199]; Squire [200]). It is evident from the descriptions of PTSD patients' recollections of their traumatic experiences that traumatic memories are not equivalent to the complete loss of declarative memory processing that occurs in patients with temporal lobe damage. The combination of intense implicit components interwoven with fragmented declarative recollections of isolated sensory elements of the experience in traumatic memories is perhaps a unique category of memory. Nevertheless, since PTSD patients can consciously recall details of aspects of their traumatic experiences, it would appear that the hippocampus is involved, perhaps in an abnormal manner, in the formation of traumatic memories.

These three points illustrate inconsistencies in the literature as to how stress affects the hippocampus. On the one hand, a large body of research unequivocally indicates that stress interferes with cognitive and electrophysiological measures of hippocampal functioning. On the other hand, however, emotional memories, including flashbulb and traumatic memories, can have a hippocampal (conscious/declarative) component. In the next section, we present a model of stress-hippocampus interactions which addresses how hippocampal functioning can be impaired by stress, and can also be involved in the formation of emotional memories.

## 7. TEMPORAL DYNAMICS MODEL OF STRESS-HIPPOCAMPUS INTERACTIONS

We suggest that the discrepancies between theory and research on emotion, memory, and hippocampal functioning discussed in the previous section may be resolved with a thorough assessment of the literature on the influence of emotion on LTP. A critical finding in this area of research is that manipulations that produce strong emotionality in rats can actually *enhance* hippocampal LTP. This finding was first described by Seidenbecher et al. [201], who showed that water-deprived rats given access to water around the time of tetanizing stimulation exhibited an *increase* in the duration of LTP recorded in the dentate gyrus (DG). Numerous other studies have replicated and extended this finding to show that a variety of arousing experiences, such as water immersion, exposure to novel places and objects, and spatial learning occurring around the time of the delivery of tetanizing stimulation, all increased the duration of LTP in CA1 and DG (e.g., Seidenbecher et al. [202]; Frey [203]; Li et al. [204]; Straube et al. [205]; Davis et al. [206]; Almaguer-Melian et al. [207]; Uzakov et al. [208]; Ahmed et al. [209]).

A critical component of the emotion-induced enhancement of LTP involves the activation of the hippocampus by the amygdala. Electrical stimulation of the amygdala can mimic the emotion-induced enhancement of hippocampal LTP (Ikegaya et al. [210]; Akirav and Richter-Levin [148]; Akirav and Richter-Levin [211]; Frey et al. [212]; Akirav and Richter-Levin [213]), and damage to, or inactivation of, the amygdala blocks stress effects on hippocampal LTP and spatial memory (Almaguer-Melian et al. [214]; Kim et al. [154]; Korz and Frey [215]; Kim and Jung [21]). In addition, input from the hypothalamus (Nakanishi et al. [216]) and the locus coeruleus (Harley and Sara [217]; Sara et al. [218]; Kitchigina et al. [219]; Bouret and Sara [220]), via activation of *β*-adrenergic receptors (Ikegaya et al. [221]; Vermetten and Bremner [8]; Strange and Dolan [222]; Nathan et al. [189]; Hurlemann et al. [223]), as well as the dopaminergic innervation of the hippocampus from the ventral tegmental area (VTA) (Li et al. [204]; Lisman and Grace [224]) and local release of corticotropin releasing hormone (CRH; Adamec et al. [225]; Wang et al. [226]; Wang et al. [227]; Blank et al. [228]; Chen et al. [229]), all appear to contribute to the rapid stress-induced enhancement of hippocampal LTP. *These studies indicate that hippocampal mechanisms of memory storage are rapidly engaged, rather than suppressed, by an arousing and stressful experience*.

Recent work has implicated corticosterone in the stress-induced enhancement, as well as the impairment, of hippocampal synaptic plasticity. Joëls et al. have shown that brief application of corticosterone around the time of tetanizing stimulation enhanced LTP in CA1 in vitro via nongenomic activation of mineralocorticoid receptors (Karst et al. [230]; Wiegert et al. [231]). Complementary work by Ahmed et al. [209] demonstrated that brief stress transforms protein synthesis-independent LTP into a long-lasting protein synthesis-dependent form of LTP, via activation of mineralocorticoid (MR) receptors. This group also showed that stress rapidly initiated dynamic changes in gene expression (Morsink et al. [232]), and levels of cellular signaling molecules in the hippocampus, including phosphorylated mitogen-activated protein kinase 2 (pMAPK2) and calcium/calmodulin-dependent protein kinase II (pCaMKII). Conversely, stress levels of corticosterone applied for a longer period of time (>20 minutes) increased the magnitude of inhibitory components of electrophysiological activity, such as the afterhyperpolarization (Joëls and de Kloet [233]; Kerr et al. [234]; Joëls and de Kloet [235]; Karst and Joëls [236]), and suppressed the induction of LTP (Pavlides et al. [237]; Rey et al. [238]; Kerr et al. [175]; Pavlides et al. [143]; Pavlides et al. [144]; Pavlides et al. [145]; Zhou et al. [149]; Alfarez et al. [152]; Krugers et al. [155]).

Extensive research indicates, therefore, that one cannot conclude that strong emotionality or corticosterone globally enhances or impairs hippocampal functioning; work discussed above indicates that stress or corticosterone can have both effects on the hippocampus. We propose that the manner in which emotionality affects the hippocampus follows a consistent pattern: an arousing experience must occur in close temporal proximity to the delivery of tetanizing stimulation to enhance LTP. Studies in which stress blocked LTP consistently involved a substantial (>20 minutes) delay from the initiation of the stress experience before tetanizing stimulation was delivered.

The time dependency of stress or amygdala activation effects on LTP was demonstrated directly in a series of studies by Akirav and Richter-Levin [148, 211, 213]. These investigators showed that stimulation of the amygdala 30 seconds, but not 1 hour, prior to perforant path stimulation of the hippocampus enhanced LTP in the DG. Similar findings were reported by Abe's group (Ikegaya et al. [239]; Ikegaya et al. [240]). In our studies in which stress blocked the induction of PB potentiation in vivo and in vitro (discussed above), tetanizing stimulation was always delivered at least 1, and as many as 4, hour after the stress manipulation began. Overall, these findings indicate that for a relatively brief period of time, stress or amygdala activation enhances the induction of hippocampal LTP, followed by a later developing phase when the induction of LTP is suppressed.

**Figure 3:**
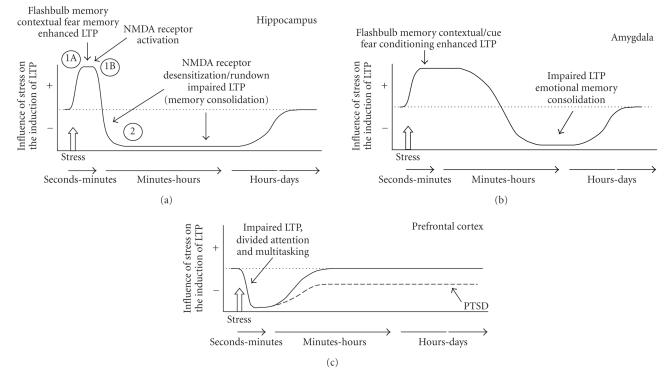
Temporal dynamics model of how stress affects memory-related processing in the hippocampus, amygdale, and prefrontal cortex. The initiation of a strong emotional experience activates memory-related neuroplasticity in the hippocampus and amygdala, and suppresses PFC functioning (phase 1). The most rapid actions would involve increases in ACTH, CRF, NE, acetylcholine, dopamine, and changes in GABA receptor binding (phase 1A), followed within minutes by elevated levels of glucocorticoids (phase 1B). The combination of the activation of the hippocampus by these neuromodulators with coincident tetanizing stimulation produces a great enhancement of LTP. Within minutes of the initiation of phase 1, the hippocampus undergoes a reversal of its plasticity state, based, in part, on the reduction in the sensitivity of NMDA receptors (phase 2). Tetanizing stimulation delivered to the hippocampus during phase 2 will thereby result in an impairment of the induction of LTP. The amygdala continues in its form of phase 1 longer than the hippocampus, but eventually, the amygdala, as well, exhibits an inhibitory phase, perhaps as it is involved in the consolidation of the emotional memory. The PFC is only inhibited by stress; the recovery from its suppression of functioning would depend on the nature and intensity of the stressor, interacting with the ability of the individual to cope with the experience. In the case of trauma-induced PTSD, the PFC may not recover to its original state of efficiency in suppressing the activity of lower brain areas, such as the amygdala and brain stem nuclei.

Figure 3 represents the temporal dynamics model, which illustrates our hypothesis that stress initiates dynamic time-restricted shifts in the efficacy of hippocampal functioning (as well as the amygdala and PFC, which are discussed in subsequent sections). This model is consistent with and extends recent theorizing by Joëls et al. [241] on the time-dependent effects of stress and corticosterone on memory and LTP, and the “emotional tagging” hypothesis of Richter-Levin and Akirav [19, 20], which states that there is a time-dependent activation, followed by inhibition, of neuroplasticity in the hippocampus in response to stimulation of the amygdala. Our model is also an extension of findings which have shown that strong emotionality briefly activates hippocampal mechanisms of synaptic plasticity, thereby increasing the duration of LTP when emotionality and tetanizing stimulation coincide in time (Ahmed et al. [209]; Reymann and Frey [242]). We emphasize more broadly in our model that stress, or any sufficiently arousing experience, briefly enhances the memory processing features of hippocampal functioning. We further speculate that this relatively brief stress-induced enhancement of hippocampal functioning underlies the declarative component of flashbulb and traumatic memories in people, and contextual fear conditioning in rodents. Following the brief period in which hippocampal plasticity is activated is a refractory period, in which there is an increase in the threshold for the induction of new plasticity. Therefore, tetanizing stimulation delivered during the poststress refractory period is less effective at inducing LTP than if it is delivered at the onset of a stress experience.

According to the temporal dynamics model, the onset of an emotional experience activates endogenous forms of neuroplasticity in the hippocampus for a period of seconds to minutes, which is revealed as an enhancement of LTP when tetanizing stimulation occurs in this narrow-time window (Ahmed et al. [209]; Reymann and Frey [242]). The activational period, identified by the “1A” and “1B” in Figure 3, involves a stress-induced increase in glutamatergic transmission and activation of AMPA and NMDA receptors (Bagley and Moghaddam [243]; Venero and Borrell [244]; McEwen et al. [245]; Kole et al. [246]). The initial component (1A) would involve the rapid activation of the hippocampus by the amygdala, in conjunction with local increases in levels of neuromodulators, such as corticotrophin-releasing hormone (CRH) (Adamec et al. [225]; Wang et al. [226]; Wang et al. [227]; Blank et al. [228]; Chen et al. [229]), acetylcholine (Ye et al. [247]; Ovsepian et al. [248]), dopamine (Li et al. [204]; Lisman and Grace [224]; Ahmed et al. [209]; Lemon and Manahan-Vaughan [249]), and norepinephrine (Gray and Johnston [250]; Hopkins and Johnston [251]; Katsuki et al. [252]; Izumi and Zorumski [253]), all of which have been shown to enhance hippocampal LTP. Rapid alterations in GABA receptor binding dynamics (Trullas et al. [254]), as well, would contribute to the almost immediate activation of the hippocampus in response to the onset of a strong emotional learning experience.

It is noteworthy that the initial component of the stress-induced activation of the hippocampus would *not* include a corticosteroid influence. The substantial delay after the onset of stress before corticosteroids would be released into the bloodstream and then reach the brain (Cook [255]) would make he steroidal modulation of hippocampal plasticity a delayed component of phase 1, identified by the “1B” in Figure 3. Thus, no sooner than several minutes after the onset of a stress experience, corticosterone would begin to activate mechanisms involved in hippocampal plasticity, thereby producing an enhancement of LTP (and memory) via nongenomic activation of mineralocorticoid receptors (Karst et al. [230]; Wiegert et al. [231]).

Ultimately, the rapid stress-induced activation of the hippocampus by steroidal and nonsteroidal neuromodulators would produce a dramatic increase in intracellular calcium levels (Kole et al. [256]; Joëls [257]; Joëls et al. [258]). This rapid influx of calcium would trigger the initiation of a cascade in the phosphorylation of molecules involved in synaptic plasticity and in the formation of memories of the events that had occurred in phase 1 (Blair et al. [259]; Poser and Storm [260]; Lisman et al. [261]; Rongo [262]; Suenaga et al. [263]).

The next phase, identified by the “2” in Figure 3, is a prolonged period of time in which the threshold for the induction of LTP is increased. When the hippocampus is in phase 2, its capacity to generate new plasticity, and therefore to form new memories, would be impaired. In theory, phase 2 can develop within minutes of the onset of a strong emotional experience, and may last from hours to days (Garcia et al. [146]; Shors et al. [264]). The initiation of phase 2 would involve the desensitization (Zorumski and Thio [265]; Rosenmund et al. [266]; Swope et al. [267]; Nakamichi and Yoneda [268]) or rundown (Rosenmund and Westbrook [269]; Alford et al. [270]; Price et al. [271]) of NMDA receptors, which occurs in response to a dramatic increase in postsynaptic calcium concentation.

The magnitudes and durations of phases 1 and 2 are variable, and would depend on the intensity and duration of the emotional experience. A weak stimulus that produces a negligible phase 1 response, as well as a weak hormonal response, would produce minimal activation of endogenous hippocampal plasticity, and thereby result in poor memory (Sandi et al. [79]). By contrast, activation of the hippocampus in phase 1 in conjunction with elevated levels of adrenal hormones (e.g., epinephrine and corticosterone) during phase 2 would facilitate the consolidation of the emotional memory. This component of the temporal dynamics hypothesis is consistent with a vast literature which has demonstrated that epinephrine- or corticosteroids- (Gold and Van Buskirk [272]; Sandi et al. [79]; McGaugh and Roozendaal [273]; Cahill and Alkire [274]; Sandi [275]; McGaugh [88]; Akirav et al. [276]; Hui et al. [85]; Roozendaal et al. [227]) administered posttraining under weak learning conditions can strengthen the consolidation of a memory that might otherwise not have been stored. Therefore, during phase 2, adrenal hormones, as well as other neuromodulators, are involved in the consolidation of information that was acquired during phase 1.

The idea that the threshold for LTP induction is raised in phase 2, rather than there being a complete suppression of hippocampal plasticity, has important functional considerations. We have commented previously that stress appears to reduce the efficiency of hippocampal processing, but does not produce the equivalent of a hippocampal lesion (Diamond et al. [52]; Diamond and Park [177]). Empirical support for this idea is the finding that, unlike stress, hippocampal lesion or inactivation produces a general impairment of spatial learning and memory in rats (O'Keefe and Nadel [278]; Olton et al. [279]; Steele and Morris [280]; Diamond et al. [52]; Morris et al. [281]; Nakazawa et al. [282]). For example, we showed that stress impaired memory in a task that placed a great demand on spatial working memory capacity, but stress had no effect on a less demanding, but still hippocampus-dependent, version of the same task (Diamond et al. [52]). Moreover, in electrophysiological studies, stress or stress-related neuromodulators have been shown to block LTP produced by relatively weak (primed burst or theta burst) tetanizing stimulation, but stress has been shown to have no effect on LTP produced by stronger forms of tetanizing stimulation (Corradetti et al. [283]; Mesches et al. [51]; Diamond et al. [139]; Alfarez et al. [152]; Vouimba et al. [140]). We interpret these findings to indicate that while the hippocampus is in the phase 2 state, it can process new information and generate plasticity, but it does so at a reduced level of efficiency. Additional support for this speculation is the finding that when the hippocampus is in a phase 2 state, it shifts to non-NMDA receptor-, rather than NMDA-receptor-, dependent LTP (Krugers et al. [155]; Wiegert et al. [156]).

The temporal dynamics model is consistent with the strong evidence, reviewed in the previous sections, that led researchers to conclude that the hippocampus is rendered “dysfunctional” or “shut down” by stress. We suggest that the idea that the hippocampus is impaired by stress was based entirely on research in which tetanizing stimulation or learning occurred while the hippocampus was in the poststress refractory period (phase 2).

In summary, we have reviewed literature which indicates that the onset of stress activates the hippocampus, thereby producing a rapid and dramatic increase in levels of intracellular calcium. The increased calcium serves as the trigger stimulus to briefly produce an enhancement (phase 1), followed by an impairment (phase 2), of the induction of endogenous synaptic plasticity in the hippocampus. Although the initiation of phase 2 is theorized to involve a calcium-triggered reduction in the sensitivity of NMDA receptors, its maintenance over hours to days may involve depotentiating mechanisms as well (Xu et al. [284]; Rowan et al. [285]; Zhuo et al. [286]; Ghetti and Heinemann [287]; Adamec et al. [288]; Lin et al. [289]; Manahan-Vaughan and Kulla [290]; Kemp and Manahan-Vaughan [117]; Gerges et al. [291]; Xia and Storm [292]; Diamond et al. [3]; Aleisa et al. [293]).

## 8. EMPIRICAL SUPPORT FOR THE MODEL

The temporal dynamics model of hippocampal functioning leads to specific predictions. First, hippocampus-dependent learning occurring coincident with the onset of an emotional experience (phase 1, Figure 3) should produce intact memory. Emotionality should rapidly activate, that is, prime, mechanisms involved in hippocampal plasticity, thereby enabling memory formation occurring while the hippocampus is in phase 1 to be intact or enhanced. Second, hippocampus-dependent memory formation should be impaired if new learning occurs during phase 2 (Figure 3).

**Figure 4:**
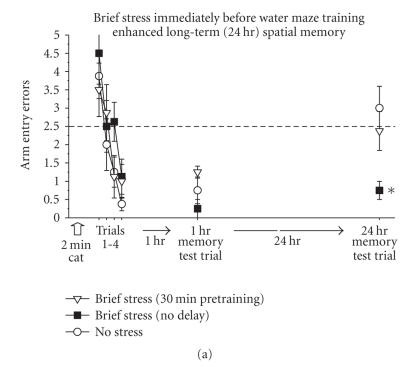

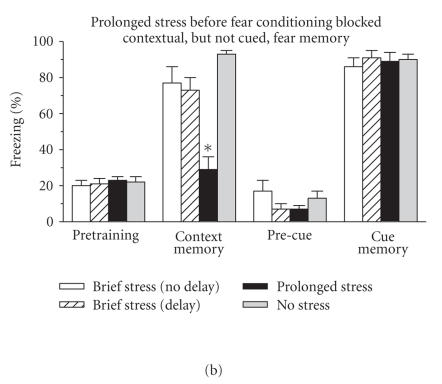
Brief stress immediately before training enhances, and prolonged stress impairs, hippocampus-dependent memory. (a) illustrates the influence of 2 minutes of predator exposure on spatial memory. Rats were exposed to a cat for 2 minutes and then they were given minimal radial arm water maze training (4 trials to find the hidden platform) either immediately or 30 minutes later. Rats trained under nonstress conditions or with cat exposure 30 minutes before training showed no evidence of memory for the platform location 24 hours later (open circle and open triangle). In contrast, rats trained immediately after brief exposure to a cat showed strong 24-hour memory (filled square). The dashed line at 2.5 errors indicates chance level of performance. (b) illustrates the effects of brief versus prolonged water immersion on contextual and cued fear conditioning. Rats given brief water stress either immediately (open bar) or 8 minutes (diagonal lines) before fear conditioning exhibited intact contextual and cued fear memory which was equivalent to that found in the no-stress group (gray bar). Rats given repeated pretraining water immersions (solid bar), by contrast, exhibited intact cued fear memory, but had a complete absence of contextual fear memory. “Precue” indicates baseline freezing in the nonshock context (3-minute duration) prior to the delivery of the tone (3-minute duration). Prolonged pretraining stress, therefore, completely suppressed contextual (hippocampus-dependent) fear conditioning without having any effect on cued (amygdala-dependent) fear conditioning. In both graphs, ∗ = *P* < .05 (ANOVA and Holm-Sidak post-hoc test) compared to the no-stress group.

We have begun to test aspects of the temporal dynamics hypothesis with two different, but well-established, tests of hippocampus-dependent memory. In the first test, adult male rats were trained in the radial arm water maze according to methods we have described in recent publications (Sandi et al. [136]; Diamond et al. [137]). In brief, rats were handled for three days and then they were given a single session of water maze training to find a hidden platform located in 1 of 6 swim arms. The rats were given only 4 sequential training trials to learn the location of the hidden platform (1 minute maximum swim time/trial, followed by 15 seconds on the platform). After completion of the four learning trials, all rats were given memory test trials 1 and 24 hours later. Results from the control (no stress) group showed that 4 learning trials were a sufficient amount of training to produce good performance on the 1-hour memory test, but was insufficient to produce good performance on the 24-hour memory test (Figure 4(a)).

According to the temporal dynamics model, the weak memory at 24 hours produced by minimal water maze training should be strengthened if training were to occur during phase 1, but not if training was to occur during phase 2. To evaluate this possibility, rats were placed for 2 minutes near a cat within the cat's housing room, as described previously (Mesches et al. [51]; Diamond et al. [52]; Woodson et al. [135]; Vouimba et al. [140]; Diamond et al. [137]; Park et al. [138]). The rats were then brought to the main laboratory, where they were given minimal water maze training, either immediately or 30 minutes later. In theory, the brief exposure of the rat to a cat should rapidly initiate an activational (phase 1) response in the rat's hippocampus. This activational phase should be followed a sufficient time later (e.g., 30 minutes) by an inhibitory (phase 2) response. Therefore, rats given water maze training immediately, but not 30 minutes, after brief exposure to a cat, should exhibit enhanced long-term spatial memory.

We have found that rats given 2 minutes of cat exposure immediately before minimal water maze training demonstrated strong spatial memory 24 hours later (Figure 4(a)). This observation of a predator stress-induced enhancement of memory is in complete contradistinction to our prior findings that exposing rats to a cat impaired their consolidation, as well as retrieval, of spatial memory (Diamond et al. [52]; Woodson et al. [135]; Sandi et al. [136]; Diamond et al. [137]; Park et al. [138]). The critical differences between the methodology of our prior studies and the current one are that here, predator stress was brief (2 minutes versus 30–60 minutes) and, more importantly, the brief stress occurred immediately before the learning phase. Therefore, 2 minutes of predator stress enhanced 24-hour memory only when it occurred immediately, but not 30 minutes, before training (Figure 4(a)).

It is important to point out that brief cat exposure enhanced the rat's memory for the location of the hidden platform, despite the fact that predator stress occurred in a completely different context from where spatial learning occurred. That is, predator stress occurred in the cat housing room and water maze training occurred in a different room. This finding does not support the theorizing of Joëls et al. [241], who stated that memory will be facilitated only for cues occurring in both the time and space in which stress occurs. The predator stress-induced enhancement of water maze memory indicates that time, but not space, is the critical element in determining which features of the stress experience will be remembered. Cues that are the focus of attention while the hippocampus is in phase 1, independent of whether they are in or out of the stress context, will be given priority for access to long-term memory storage.

This experiment leads to one other prediction. Since we hypothesized that exposure of the rats to the cat should drive the hippocampus into a phase 1 state of enhanced plasticity, then the rats also should have a strong memory of their cat exposure experience. In the water maze-cat exposure experiment (described above), the memory of the rats' exposure to the cat was not measured, but in other work, we have found that rats develop a strong, extinction-resistant, fear of the context temporally associated with their exposure to the cat (Halonen et al. [294]). This preliminary finding provides further support for the idea that the hippocampus is powerfully activated by traumatic stress to form a durable memory of the arousing experience, as well as other, temporally contiguous, experiences.

In theory, once the phase 1 activational “window” closes, and phase 2 begins, the hippocampus becomes less efficient at processing new information. Therefore, 30 minutes after cat exposure occurred, the hippocampus would have been less efficient at storing the memory of the platform location, which explains why rats given minimal water maze training 30 minutes after cat exposure had poor memory for the platform location 24 hours later.

We have conducted a second test of the temporal dynamics hypothesis by examining the influence of pretraining stress on new learning occurring when the hippocampus presumably was in phase 2, which is a time when we would expect that memory formation (for phase 2 events) should be impaired. It is well known that hippocampal damage or inactivation can interfere with contextual, but not cued, fear conditioning (Phillips and LeDoux [181]; Maren [182]; Sanders et al. [183]; Rudy et al. [184]). Therefore, we hypothesized that an impairment of contextual (hippocampus-specific) memory should occur if fear conditioning were to occur when the hippocampus was driven into the phase 2 state.

Adult male Sprague-Dawley rats (*n* = 8/group) were given 1 (brief stress) or 10 (prolonged stress) inescapable immersions in a tank of water (1.7 m diameter, 30 cm depth, 23-24°C). Two groups of rats were given a single water stress (1 minute of water immersion) and then they were given fear conditioning training either immediately (brief stress-no delay) or 8 minutes later (brief stress-delay). The group of rats given prolonged water stress swam for an average of 35 seconds per immersion, followed by a 15-second period out of the water, which was repeated 10 times in an 8-minute period. After the tenth immersion in water, the rats in this group were immediately given fear conditioning training (prolonged stress).

Fear conditioning training was designed in order to produce strong contextual and cued fear memory. Rats were placed into a conventional shock box for 2 minutes, followed by the delivery of 10 shocks (1 mA for 2 seconds) pseudorandomly delivered over 30 minutes (the range of time between shocks was 2–4.5 minutes, with an average delay of 3 minutes). Before each of the 10 shocks, a tone was delivered for 10 seconds, with the last 2 seconds of the tone coincident with the delivery of shock. Twenty four hours after training, all rats were reexposed to the shock environment for 5 minutes for the contextual fear memory test and then they were placed in a different environment where the auditory cue was delivered for 3 minutes. Conditioning was measured as the percent of time that the rats exhibited immobility (freezing) to the context or cue, as determined by automated detection of their movement (Coulbourne instruments).

The rats that were given a single 1-minute immersion in the water immediately before fear conditioning was expected to exhibit intact contextual fear memory because brief water exposure would be expected to drive the hippocampus into the phase 1 state. In contrast, the rats that experienced repeated immersions in the water were expected to exhibit impaired contextual fear memory because more prolonged stress would be expected to drive the hippocampus into the phase 2 state (Figure 3).

We have found that rats given brief pretraining water stress immediately before fear conditioning exhibited contextual and cued fear conditioning which was equivalent to the degree of conditioning observed in the nonstressed group (Figure 4(b)). Therefore, brief stress occurring immediately before fear conditioning did not adversely affect hippocampus-dependent memory processing (the fear memory under control training conditions was so strong that it was not possible to observe a brief stress-induced enhancement of the fear memory).

The memory performance of rats given prolonged water stress prior to fear conditioning training was quite different from the memory performance of rats given brief water stress. Rats given 8 minutes of pretraining stress exhibited intact cued (amygdala-dependent) fear memory, but they exhibited a complete absence of contextual (hippocampus-dependent) fear memory (Figure 4(b)). Thus, the performance of rats given prolonged pretraining stress was equivalent to the severe contextual memory impairment which has been reported in rats with an inactivated or damaged hippocampus (Phillips and LeDoux [181]; Maren [182]; Sanders et al. [183]; Rudy et al. [184]).

It is important to point out that the inhibitory effect of water stress on contextual fear conditioning was produced by the repeated immersions of the rats in the water, and not only because the water stress began 8 minutes before fear conditioning training. Rats that were given only a single immersion in the water 8 minutes before fear conditioning developed intact contextual and cued fear memory (brief stress—delay group, Figure 4(b)). This finding indicates that there is an interaction between the strength and duration of the stress experience which is necessary to drive the hippocampus into a phase 2 state.

Taken together, our findings in which brief stress enhanced water maze memory (Figure 4(a)) and prolonged stress impaired hippocampus-specific (contextual) fear memory (Figure 4(b)) support our hypothesis that stress rapidly initiates dynamic shifts (enhancement followed by inhibition) in the efficiency of hippocampal memory processing. Moreover, the fear conditioning experiment suggests that phase 2 can be initiated within 8 minutes of the onset of a stressful experience if the stress is sufficiently strong and persistent. The basis of the delayed stress-induced suppression of hippocampal processing may involve a stress-induced increase in GABAergic transmission in the hippocampus (Trullas et al. [254]; Amitani et al. [295]), in addition to an activity-induced desensitization of NMDA receptors (discussed above).

The water maze and fear conditioning findings described here are potentially relevant towards understanding the physiological basis of flashbulb memories. The relatively brief period in which the hippocampus would be activated by stress would be a sufficient time to initiate NMDA, and perhaps non-NMDA, receptor-mediated plasticity (Joëls et al. [258]; Krugers et al. [155]; Wiegert et al. [231]; Morsink et al. [232]), which would induce the hippocampus to store information about the arousing experience. However, when the hippocampus is briefly in this global activational state, its mechanisms involved in memory storage are promiscuous, storing information not only about the arousing stimulus (the “to-be-remembered” (TBR) event; Christianson [29]), but also about temporally contiguous information unrelated to the TBR event. The end product would be an emotional memory which would be a montage of significant and insignificant events that co-occurred in time. In terms of flashbulb memory processing, the activation of the hippocampus by an arousing event would initiate the storage of the memory of a TBR event, such as the televised images of planes crashing into the World Trade Center on September 11, 2001, as well as coincident information, such as where people were and what they were doing, as they learned of the crisis.

Additional empirical support for the temporal dynamics model is derived from the “warning signal” hypothesis by Ehlers et al. [198]. These investigators noted that intrusive memories in PTSD patients were typically composed of the remembrance of stimuli that were present immediately before the traumatic event happened or shortly before the moments that had the largest emotional impact. They suggested that intrusive memories are not random sensory fragments of the traumatic experience. Instead, they noted that intrusive memories “can be understood as stimuli that—through temporal association with the traumatic event—acquired the status of warning signals; stimuli that if encountered again would indicate impending danger” (page 999). Our temporal dynamics model extends their “warning signal” hypothesis to a physiological level, as we propose that it is the abnormally intense and time-restricted activation of the hippocampus in phase 1 that can produce a powerful association between coincident neutral and traumatic stimuli which is commonly described as “burnt into memory” (Elbert and Schauer [296]).

Other findings from our group are consistent with the idea that within 30 minutes after the onset of phase 1, the hippocampus undergoes a prolonged period in which the induction of new plasticity or the formation of new memories is impaired. First, we have shown that 30 minutes of cat exposure not only impaired spatial memory (Kim and Diamond [1]; Diamond et al. [10]; Diamond et al. [2]; Diamond et al. [3]), it also suppressed molecular (Sandi et al. [136]) and structural (Diamond et al. [137]) measures of plasticity in the hippocampus. Specifically, 30 minutes of cat exposure impaired spatial memory and dramatically reduced hippocampal levels of neural cell adhesion molecules (NCAMs) (Sandi et al. [136]), which are important structural components of long-term memory storage (Sandi [297]). Second, we have found that 30 minutes of pretraining cat exposure suppressed the learning-induced increase in dendritic spine density in CA1 (Diamond et al. [137]). Overall, these findings, in conjunction with related work by Kim et al. [154] support our hypothesis that a strong stressor generates a powerful inhibitory influence on hippocampal memory processing for events occurring 30–60 minutes after the onset of a stressful experience.

## 9. WHAT IS THE BENEFIT OF SUPPRESSING THE INDUCTION OF HIPPOCAMPAL PLASTICITY IN PHASE 2?

Why does the hippocampus undergo a prolonged phase of inhibition of the induction of synaptic plasticity following the activational phase? We can suggest three benefits of the phase 2 state of inhibition. First, if a stress-induced increase in hippocampal activation, with its increase in glutamate levels and enhanced calcium influx, were to continue unabated, hippocampal neurons would be at an increased risk for glutamate-induced neurotoxicity (Sapolsky [298]; Slemmer et al. [299]; Petrović et al. [300]). The decrease in the sensitivity of NMDA receptors during phase 2 would reduce calcium influx, thereby protecting hippocampal neurons from developing excitotoxicity in times of strong and persistent stress (Moudy et al. [301]; Moulder et al. [302]).

A second explanation for why the desensitization and rundown of NMDA receptors occur during phase 2 is that it serves a “memory protective” function. In theory, the activation (phase 1) followed by inhibition (phase 2) of hippocampal plasticity would produce a relatively brief period, an isolated fragment of time, when the formation of the memories of events occurring at the onset of an emotional experience would be optimized, thereby enhancing the association between otherwise neutral cues with the onset of a traumatic experience (Ehlers et al. [198]). Thus, a primary component of the neurobiology of flashbulb memories is the brief activation of neuroplasticity in the hippocampus while it is in the phase 1 state. The subsequent suppression of the induction of new plasticity from being generated in phase 2 would reduce, but perhaps not completely block, the corruption of the memory of phase 1 events by later occurring events (Laney and Loftus [93]; Loftus [303]).

Third, processes initiated during phase 1 and then active in phase 2, such as the corticosterone-mediated activation of the GR receptor, genomically mediated events, and protein synthesis, would underlie the first phase of the consolidation of the emotional memory. As hippocampal neurons proceed through the molecular sequence of events leading to structural plasticity underlying the storage of the memory of the emotional event, it would be prudent for the storage process to occur without being contaminated by the processing of new information. Therefore, as the hippocampus descends into phase 2, it goes partially “offline” for a period of hours as the hippocampus begins to consolidate information acquired during phase 1.

## 10. A PLACE FOR THE TEMPORAL DYNAMICS MODEL IN THEORIES OF HIPPOCAMPAL FUNCTIONING

Our temporal dynamics model suggests that qualitative features of hippocampal memory processing in response to stress should be different from the type of memory processing which is normally attributed to the hippocampus. That is, over the past few decades, investigators have developed the view that the hippocampus plays a role in binding together the elements of an experience to generate a “cognitive map” (O'Keefe and Nadel [278]), or a “conjunctive” (Sutherland et al. [304]; Rudy and O'Reilly [305]; O'Reilly and Rudy [306]) and flexible (Cohen and Eichenbaum [307]) representation of a learning experience. Extensive research supports these theories, indicating that the hippocampus enables the formation of “complex, bound representations of episodes replete with spatiotemporal and contextual details” (Metcalfe and Jacobs [61, page 187]). Thus, the different theories on the role of the hippocampus in memory processing have in common the idea that the hippocampus generates a higher-order representation of the contextual components of a learning experience (Teyler and DiScenna [308]; Eichenbaum [309]; O'Reilly and Rudy [306]; Brassen et al. [310]).

The extensive evidence of a stress-induced impairment of LTP and spatial memory provided strong support for the view that stress suppresses hippocampal functioning. But we suggest that another reason why the hippocampus was considered to be dysfunctional in times of emotional trauma is not only because of the stress-LTP work, but because the characteristics of traumatic memories did not conform to the well-accepted view that the hippocampus generates memories which contain a higher-order (cognitive map/conjunctive) representation of the learning context. Traumatic memories have been described as disembodied fragments of the original experience only weakly connected with contextual details (van der Kolk [176]; van der Kolk and Fisler [311]; van der Kolk [170]; van der Kolk [312]; Ehlers et al. [198]; Hackmann et al. [313]; van der Kolk [314]), which is inconsistent with the cognitive map/conjunctive view of the hippocampal representation of a learning experience. This perspective is illustrated by the following perspective by van der Kolk [170] on why the hippocampus is impaired in times of trauma:

“very high levels of emotional arousal may prevent the proper evaluation and categorization of experience by interfering with hippocampal function. One can hypothesize that when this occurs, sensory inprints of experience are stored in memory; however, because the hippocampus is prevented from fulfilling its integrative function, these various inprints of experience are not organized into a unified whole. The experience is laid down, and later retrieved, as isolated images, bodily sensations, smells, and sounds that feel alien and separate from other life experiences. Because the hippocampus has not played its usual role in helping to localize the incoming information in time and space, these fragments continue to lead an isolated existence” (page 295).

Our temporal dynamics model provides a different perspective from van der Kolk's on the possible involvement of the hippocampus in emotional and traumatic memory processing. The model proposes that in times of emotional trauma, the memory storage reportoire of the hippocampus rapidly shifts from its normative cognitive map mode to a flashbulb memory mode, which processes time-restricted, contextually disembodied, fragments of the details of emotional experiences. We hypothesize that the great enhancement and durability of memory for the details of arousing experiences is produced in part by the rapid induction of neuroplasticity in the hippocampus in phase 1 (Figure 3), mediated by arousal-related afferents, including the amygdala (Abe [18]; Roozendaal et al. [315]; Abe et al. [316]; Richter-Levin [20]; McGaugh [88]; Akirav and Richter-Levin [22]), hypothalamus (Nakanishi et al. [216]), ventral tegmental area (Ovsepian et al. [248]; Lisman and Grace [224]), and locus coeruleus (Sara and Devauges [317]; Harley and Sara [217]; Kitchigina et al. [219]).

We would also speculate that in the days, weeks, and even years after a traumatic event occurs, with repeated rehearsals of the experience, a person's hippocampus may attempt to reconstruct a more contextually rich representation of the original emotional experience (Foa et al. [318]; Diamond et al. [17]). The reconstructed memory would therefore be a hybrid representation of information processed by the hippocampus (and amygdala) in a fragmented manner at the time of the experience, in conjunction with postevent reconstructions of the memory. The repeated reconstruction, as well as reconsolidation (Przybyslawski and Sara [319]; Nader et al. [320]; Duvarci and Nader [321]), of the representation of the original experience by the hippocampus could produce a hypermnesic (strengthening) of the memory of the traumatic experience (Scrivner and Safer [322]; Klein et al. [323]; Bornstein et al. [324]; Kern et al. [325]). However, repeatedly reconsolidating the memory could render it susceptible to modification, and potentially reduce its veracity (Foa et al. [318]; Garry et al. [326]; Christianson and Lindholm [327]; Wright and Loftus [328]; Loftus [303]).

Despite the well-described evidence of the modifiability of flashbulb memories, it appears that information acquired during phase 1, which is when there would be the most intense activation of hippocampal and amygdala neuroplasticity, is highly resistant to develop reconstructive errors over time (van der Kolk et al. [329]; van der Kolk [176]; Koss et al. [330]). As noted by van der Kolk [170], “aspects of traumatic events appear to become fixed in the mind, unaltered by the passage of time or by the intervention of subsequent experience” (page 282). Thus, the “warning signal” hypothesis of Ehlers et al. [198], which emphasizes that traumatic memories commonly include events that had occurred at the onset of the traumatic experience, and the resistance of traumatic memories to corruption by later occurring events, both indicate that phase 1 of our temporal dynamics model is a period of highly efficient hippocampal processing. When the hippocampus is driven into phase 1 by strong emotionality, its focusing on events associated with emotional experiences, referred to as “tunnel memory” by Safer et al. [104] results in powerful memories of isolated sensory experiences which are extremely resistant to degradation over time. We would suggest that it is the memory for events occurring during phase 2 (Figure 3) and for events occurring outside of the focus of attention during the emotional experience that are more susceptible to corruption over time than events which were the focus of attention during phase 1 (Christianson [29]).

In summary, we have proposed that the initiation of a stressful experience produces an intense, but brief, activation of memory-encoding plasticity within the hippocampus. This process would involve a shift by the hippocampus from its normative cognitive mapping mode to a “print-now” (Brown and Kulik [37]) flashbulb memory mode. Within minutes after being activated by the emotional experience, the hippocampus would descend into the phase 2 state, which would involve an increase in the threshold for the induction of new plasticity. It is during the phase 2 state that the hippocampus would exhibit an impairment in the induction of LTP, and therefore, be impaired at storing the memory of events that occur during phase 2. Long after the termination of the emotional experience, the hippocampus would slowly return to its cognitive mapping mode and it would attempt to generate a contextually rich representation of the experience. With the hippocampus in this reconstructive phase, post-trauma experiences and ideations may become “spliced” into memories of the original events. In this manner, information stored around the time of the emotional experience may become incorporated into a more complete, but possibly corrupted, representation of the original experience (Neisser and Harsch [331]; Neisser [332]).

## 11. FLASHBULB MEMORIES AND THE STRESS-INDUCED MODULATION OF LTP IN THE AMYGDALA

It is well known that the amygdala is a critical component of emotional learning and memory. This topic has been reviewed extensively by others (LeDoux [333]; McIntyre et al. [334]; Fanselow and Gale [335]; McGaugh [88]; Dityatev and Bolshakov [336]; Maren [337]; Kim and Jung [21]; Sigurdsson et al. [338]) and will not be discussed at length here. The primary issue we are concerned with is how an emotional experience affects endogenous mechanisms of plasticity, as well as electrical stimulation-induced LTP, in the amygdala. An early study that addressed this issue was the work by Rogan et al. [339]. These investigators demonstrated that fear conditioning produced an enhancement of CS-evoked activity in the amygdala. Comparable results were reported by McKernan and Shinnick-Gallagher [340], who showed that fear conditioning produced a presynaptic facilitation of AMPA-receptor-mediated transmission, in vitro. In both studies, the increases in intrinsic excitability in the amygdala produced by fear conditioning were specific to associative processes, as shock, alone, did not produce a change in excitability. These studies, as well as subsequent work from this group (Schroeder and Shinnick-Gallagher [341]) and studies by Adamec et al. employing naturalistic (predator) stress (Adamec et al. [342]; Adamec et al. [288]; Rosen et al. [343]), all indicate that fear conditioning produces long-lasting increases in excitability in the amygdala.

As we noted in an earlier section, whether or not tetanizing stimulation induces LTP can be viewed as a “diagnostic” measure of the functioning of a brain structure. How does stress or fear conditioning affect exogenously induced LTP in the amygdala? Our group, in conjunction with Richter-Levin's group, examined this issue in recordings from the basal amygdala of behaving rats (Vouimba et al. [344]). We showed that stress exerted different effects on LTP in the DG versus the basal amygdala in response to stimulation of the entorhinal cortex. In general, stress either had no effect or suppressed LTP in the DG, and enhanced LTP in the basal amygdala. In more recent work, our group has shown that predator stress blocked PB potentiation in CA1 and enhanced LTP in the basolateral nucleus of the amygdala (Vouimba et al. [140]). These studies suggest that when the hippocampus passes into the phase 2 (inhibitory) period, the amygdala continues to exhibit a stress-induced enhancement of plasticity (Figure 3).

The finding of an enhancement of LTP in the amygdala under stress conditions is consistent with the well-established role this structure serves in emotional memory. There are, however, accounts in which amygdala LTP has been suppressed in response to emotional learning conditions. For example, Tsvetkov et al. [345] found that 3 days of fear conditioning resulted in a profound suppression of LTP in the cortico-amygdala circuit, and Schroeder and Shinnick-Gallagher [341] found a suppression of amygdala LTP 10 days after fear conditioning. Comparable findings were reported recently by Kavushanky et al. [346], who showed that rats given water maze training exhibited a reduction in the magnitude of LTP in the basal amygdala in response to tetanizing stimulation of the EC. The findings of an emotional learning-induced suppression of LTP in the amygdala suggest that this structure, as with the hippocampus, has an initial activational phase of processing, followed by a slowly developing inhibitory phase. The amygdala appears to remain in phase 1 longer than the hippocampus, but eventually, the phase 2 (inhibitory) period develops, perhaps while the amygdala is involved in the consolidation of the emotional memory (Izquierdo and Medina [347]; Pelletier and Paré [348]; McGaugh [88]).

We should emphasize that the amygdala excitability curve in Figure 3 serves only to illustrate our idea that the amygdala, as with the hippocampus, appears to undergo activational and inhibitory phases which may be involved in the consolidation of emotional memories. The actual shapes of perhaps multiple plasticity-shift curves in different amygdala nuclei would reflect interactions between activational and inhibitory influences in response to an emotional experience. Despite these caveats, our model is potentially useful in providing insight into the neurobiology of emotional, in particular flashbulb and traumatic, memories. For example, because the model indicates that the amygdala and hippocampus each develops endogenous plasticity independently with the onset of a stressful learning experience, there should be distinguishable hippocampal versus amygdaloid components of flashbulb memories. This feature of the model is consistent with almost a century of observations of people with organic, as well as emotion-induced, memory disorders. One example is a well-known case study of an amnesic patient, presumably with hippocampal damage, studied by Claparède [349]. He conducted an experiment in which he shook the patient's hand, and at the same time, stuck her with a pin which was hidden between his fingers. The patient, some time later, exhibited a reluctance to shake his hand, but she did not have a specific recollection of the handshake/pin prick incident (translated to English in Claparède [349]). Similarly, Bechara et al. [350] reported that a patient with bilateral damage to the hippocampus failed to make a CS-US association at a cognitive (explicit) level, but did develop a subconscious CS-US association. Conversely, another patient with damage to the amygdala given fear conditioning failed to develop a conditioned emotional response, but did learn the factual (explicit) information about the CS-US contingency. Finally, a patient with bilateral damage to the hippocampus and amygdala failed to acquire either the explicit details or a conditioned emotional response. These cases are only a subset of a substantial literature consistent with the idea that the hippocampus and amygdala process different features of emotional memories (Phillips and LeDoux [351]; LeDoux [352]; Bechara et al. [350]; Fanselow [353]; Sanders et al. [183]; Bechara et al. [354]).

One other case is particularly instructive towards understanding how the amygdala and hippocampus process different components of emotional (traumatic) memories, with potential relevance towards understanding the etiology of post-traumatic stress disorder (PTSD). Krikorian and Layton [355] reported on a case of a healthy adult man who was rendered anoxic for approximately 15 minutes when he was suddenly buried under 5.5 meters of sand. In the weeks following his recovery, he exhibited a change of personality, which was presented largely as persistent cognitive impairments and symptoms of PTSD. He spent his days with a near- constant fear of imminent death and intrusive thoughts that the earth would open up and swallow him, and his nights were consumed with nightmares about being buried alive. Despite these powerful PTSD-like symptoms which could be directly tied to his traumatic experience, he had no recollection of the actual event.

We suggest that the initiation of the burying incident triggered a powerful activation of neuroplasticity simultaneously in his hippocampus and amygdala. The independent induction of plasticity in each of these two structures would normally function to form a flashbulb memory which would contain two components: (1) the explicit, hippocampus-dependent, information about the specific details of the experience; (2) more global, conscious, and subconscious, amygdala-dependent components which would generate the fear-provoking features of the memory. However, because the man remained in an anoxic state for so long, it is likely that he developed damage to his hippocampus (Zola-Morgan et al. [356]; Squire and Zola [357]; Rempel-Clower et al. [358]), which interfered with the consolidation of the explicit component of the memory of his traumatic experience. The cognitive deficits this patient exhibited post-trauma are consistent with our assumption that he developed hippocampal damage as a result of his anoxia. We would speculate that global and fear-provoking information about the experience was stored primarily by amygdala-centered memory processing, thereby underlying his general fear of being buried and his PTSD symptomology. This postulated role of the amygdala in the gist, rather than the details, of an emotional experience is consistent with recent findings (Adolphs et al. [359]; Cahill and van Stegeren [360]) and discussion (Phelps [361]) of the differential roles of the hippocampal versus amygdala in emotional memory processing.

In summary, findings from amnesics, in conjunction with observations of people with emotional trauma-induced amnesia, support our hypothesis that the hippocampus and amygdala both develop neuroplasticity in the seconds to minutes after the initiation of a traumatic experience. The engram of the resultant flashbulb memory is therefore a montage of hippocampal and amygdala representations of the experience.

## 12. STRESS TAKES THE PREFRONTAL CORTEX “OFFLINE”

In 1898, Overton [362] proposed that “Thinking is done by the cells of the brain behind the forehead … if the forehead cells do not know how to think, the mind cannot make use of memories. We say that such a person is a fool, even though he has great knowledge.”

A century later, Arnsten [363] stated that “stress impairs prefrontal cortex function through catecholamine receptor mechanisms … dopamine and norepinephrine synergize to take the prefrontal cortex “off-line” during stress.”

The functioning of the PFC, and its susceptibility to be disrupted by stress, is aptly summarized by the two statements above by Overton [362] and Arnsten [363]. “Thinking,” or higher-order cognitive functioning, is dependent to a great extent on the integrity of the PFC. Extensive research and recent imaging studies have shown that the PFC is critically involved in guiding behavior during divided attention (Nebel et al. [364]; Dannhauser et al. [365]) and working memory (Goldman-Rakic [366]; Adcock et al. [367]; Taylor et al. [368]; Marshuetz and Smith [369]; Müller and Knight [370]; Curtis [371]) tasks, as well as in planning (Rowe et al. [372]; Anderson et al. [373]) and decision making (Bechara [374]; Bechara [375]), which may be broadly referred to as “executive processes” (Baddeley and Della Sala[376]; McEwen [377]). In addition, the frontal cortex, in general, is an important component of brain circuitry involved in the extinction of conditioned responses (Maren and Quirk [378]; Likhtik et al. [379]; Milad et al. [380]; Milad et al. [381]), behavioral inhibition (Tillfors [382]; Levy [383]), and coping with controllable stressors (Ter Horst [384]; Gerrits et al. [385]; Rangel et al. [386]; Bland et al. [387]; Amat et al. [388]), as well as in interacting with the temporal lobe to faciltate memory formation and retrieval (Buckner and Wheeler [389]). Therefore, Overton's statement about cells at the front of the brain being involved in “thinking” is accurate in the sense that the PFC (and other frontal and parietal regions) is important for higher-order attentional and cognitive processes which enable an individual to use information and memory effectively. Foolish behavior, such as poor decision making, is well known to occur when frontal cortex functioning is impaired as a result of damage (Bechara et al. [390]; Bechara [374]; Bechara [375]) or acute stress (Arnsten and Goldman-Rakic [391]; Arnsten [392]; Arnsten [393]; Gray [394]; Morrow et al. [395]; Arnsten [396]; Moghaddam [397]; Birnbaum et al. [398]; Moghaddam and Jackson [399]; Goudriaan et al. [400]).

With regards to LTP work, we are aware of only two studies that have investigated how acute stress affects LTP in the PFC. Maroun and Richter-Levin [401] showed that electrical stimulation of the amygdala produced LTP in the PFC. These researchers demonstrated that the same stress that blocked LTP in CA1 (placement of rats on an elevated platform) also blocked LTP in the PFC. Similarly, Rocher et al. [402] demonstrated that LTP in the PFC produced by stimulation of the ventral hippocampus was blocked by elevated platform stress.

The inhibition of LTP in the PFC by stress, acting in large part, through excessive activation of dopamine (D1) receptors, supports the idea that PFC functioning, in general, including its capacity to maximize decision making, multitasking, and divided attention, is impaired by stress (discussed above). Therefore, we have illustrated a rapid and prolonged inhibitory shift in functional excitability in the PFC in our model of stress-LTP dynamics (Figure 3). This inhibitory phase of PFC functioning would be revealed electrophysiologically as a suppression of LTP, and behaviorally as an impairment of coping skills, executive functioning, multitasking, decision making, and a reduced ability to perform well in complex tasks.

The length of time it would take for the stress-induced inhibition of PFC functioning to recover fully to baseline would depend on the nature and intensity of the stressor, interacting with environmental and genetic factors, as well as with individual variability in coping effectively with the stressor (Yehuda [403]; Olff et al. [404]; Nemeroff et al. [102]). In extreme cases, individuals who develop PTSD in response to experiencing a traumatic event may be unable to recover fully to their original baseline (Figure 3). The ongoing impairment of PFC functioning would result in a chronic reduction in descending inhibitory influences from the PFC on brainstem nuclei and the amygdala (Williams et al. [405]), which could form the basis of certain symptoms of PTSD, such as chronic hypervigilance, attention deficits, and impaired executive functioning (Vermetten and Bremner [406]; Shin et al. [407]; Britton et al. [408]; Shin et al. [409]; Williams et al. [405]).

## 13. STRESS EFFECTS ON THE PFC, HIPPOCAMPUS, AMYGDALA, AND THE YERKES-DODSON LAW

The relationship between stress effects on the PFC, hippocampus, amygdale, and the Yerkes-Dodson law has been alluded to throughout this paper. For example, we have emphasized how the PFC (and related frontal areas) is involved in complex tasks that require working memory, executive processing, decision making, and divided attention. Therefore, the extent to which the PFC is involved in a task and the degree to which the PFC is suppressed by emotionality are primary determinants of whether a task's arousal-performance curve will be linear or curvilinear. That is, if the successful completion of a task requires PFC functioning, then performance on that task is likely to suffer under conditions of high arousal. One example of an application of this strategy is the finding that high states of anxiety have little to no effect on performance in simple, single-digit, mental calculations, which place minimal demands on PFC-based working memory capacity. Ashcraft [410] has shown that when people perform more complex mental calculations, such as double-digit calculations, which tax working memory and thereby increase PFC involvement in the task, they are more susceptible to be impaired by anxiety. It is notable that even single-digit calculations could be made susceptible to impairments by anxiety when a PFC-dependent component, decision-making, was included in the calculations (Ashcraft [410]). Therefore, one strategy with which to operationalize the distinction between “simple” and “complex” tasks is to determine whether the task involves a PFC-mediated component. We would suggest that, as a general rule, tasks that require the involvement of the PFC, which can be confirmed to some degree by neuroimaging techniques (Callicott et al. [411]; Ranganath et al. [412]; Taylor et al. [368]; Ranganath and D'Esposito [413]; Curtis [371]), should all exhibit the curvilinear component of the Yerkes-Dodson law.

The mechanistic basis of the PFC-mediated curvilinear component of the Yerkes-Dodson law is well studied. A number of researchers have commented on the inverted-U-shaped relationship between dopamine receptor signaling in the prefrontal cortex and working memory performance (Arnsten et al. [414]; Murphy et al. [415]; Cai and Arnsten [416]; Arnsten [363]; Arnsten [417]; Brunel and Wang [418]; Dreher et al. [419]; Yamashita and Tanaka [420]; Williams and Castner [421]; Tanaka et al. [422]). The common finding among these studies is the importance of an intermediate, that is, optimal, level of dopaminergic (D1) receptor activation to enable working memory tasks to be accomplished. Stress, pharmacological treatments, or mental disease states (Russell [423]; Levy [383]; Jay et al. [9]; Anderson et al. [424]) that involve either an excessive increase or decrease in dopaminergic activity result in an impairment in working memory performance (Arnsten [363]; Williams and Castner [421]).

An inverted-U function has also been described for the relationship between locus coeruleus (LC) activity and performance in an attentional task (Aston-Jones et al. [62]; Aston-Jones et al. [64]). In the work by Aston-Jones' group, behavioral performance was impaired in animals with high levels of LC activity, perhaps because the task required sustained attention with distracting stimuli. Overall, there is strong support for the idea that intermediate levels of norepinephrine and dopamine in the PFC are an important component of efficient performance on complex tasks (Arnsten [363]; Williams and Castner [421]).

The second component of the Yerkes-Dodson law is the enhancement of performance under high levels of stress in relatively simple tasks (Figure 2(b)). If, for example, a task involves focused attention to an isolated cue with minimal cognitive (decision-making) demands, then performance may not only be unimpaired, it can even be enhanced, under conditions of high arousal. The well-described “weapon-focus” phenomenon, as well as fear conditioning in rats, illustrates a situation that involves an almost complete absence of decision making, multitasking, and peripheral attention (Christianson [29]; Conway et al. [425]; Safer et al. [104]; Pickel [105]). In threatening situations, there may be a great enhancement of memory for the sole focus of attention, such as the weapon that threatened someone's life, with perhaps impaired memory for other cues on the periphery of a person's attention (Christianson [29]; Safer et al. [104]; Pickel [105]). This shift in focus from thoughtful decision making to one of highly focused attention with rapid processing has clear adaptive value, enabling an individual to devote attentional resources (and maximal hippocampal and amygdaloid memory processing) to life-threatening stimuli in times of danger (Mineka Öhman [426]; Flykt [427]).

As a first step in understanding how emotion enhances learning in simple tasks, consider the repercussions of the suppression of the PFC by strong emotionality. Descending projections from the PFC appear to provide an inhibitory influence over lower brain structures involved in emotionality, such as the amygdala, dorsal raphe and hypothalamus (Arnsten and Goldman-Rakic [428]; Sesack and Pickel [429]; Rempel-Clower and Barbas [430]; Hajós et al. [431]; Quirk and Gehlert [432]; Quirk et al. [433]; Milad et al. [434]; Likhtik et al. [379]; Amat et al. [388]). A consequence of the loss of PFC-mediated inhibition is that these structures will exhibit greater activation in times of strong emotionality, thereby enhancing their throughput. For example, the release of PFC-mediated inhibition over locus coeruleus cell activity will increase norepinephrine release throughout the forebrain, which would be manifested behaviorally as an enhancement of attention, and physiologically as enhanced memory-related neuroplasticity in the amygdala and hippocampus (Izquierdo and Medina [435]; Roozendaal [436]; McGaugh [437]; Strange and Dolan [222]; Hurlemann et al. [223]; Bremner [100]). Indeed, we would speculate that it is the release of PFC inhibition over brain stem and amygdala activity which would enable the great enhancement and focusing of attention towards threatening cues (Berridge et al. [438]).

Finally, errors in emotional memory processing are not attributable solely to an impairment of PFC function. Flaws in emotional memories have been a subject of extensive research, which has great relevance in clinical and legal settings, involving issues including, for example, the credibility of repressed memories (Loftus [439]; Loftus and Polage [440]) and eyewitness testimony (Loftus [441]; Sparr and Bremner [422]). Elsewhere, we have commented on the functional consequences of how acute stress appears to simultaneously enhance plasticity in the amygdala and impair plasticity in the hippocampus (Vouimba et al. [140]). One potential repercussion of the opposing effects of stress on these two structures is that in times of strong emotionality, amygdala plasticity is enhanced, thereby intensifying the emotional memory of an experience. However, if the enhancement of the amygdala processing occurs at a time when the hippocampus is in the stress-induced inhibitory period (Figure 3, phase 2), then the stress-induced impairment of hippocampal functioning could compromise the accuracy of the details of the emotional memory, despite an individual's great confidence in its veracity (Talarico and Rubin [443]; Wolters and Goudsmit [444]; Coluccia et al. [445]). Therefore, in addition to the reduced involvement of the PFC in controlling cognition in times of strong emotionality, reduced functioning of the hippocampus while it is in the phase 2 state, as well, contributes to the impairment of performance at the right side of the curvilinear component of the Yerkes-Dodson law.

In conclusion, a century after the passage of the Yerkes-Dodson law and almost 50 years after the publication of Easterbrook's cue utilization hypothesis, cognitive psychology and behavioral neuroscience research have provided an in-depth perspective on the neurobiological basis of how emotion interacts with memory formation. We have applied this research to develop a synthesis which addresses the linear and curvilinear components of the Yerkes-Dodson law. We have proposed that the enhancement of memory under high stress conditions is subserved by the rapid and coordinated activation of hippocampal-amygdaloid circuitry, in conjunction with a suppression of the PFC. The emotional-induced enhancement of hippocampal and amygdaloid processing favors rapid processing of distinct cues with minimal demands on decision making, which is typified by phenomena such as weapon focus and flashbulb memories in people and fear conditioning in rats. We have also suggested that the high (declining) end of the curvilinear component of the Yerkes-Dodson law is generated largely by a stress-induced suppression of PFC functioning (see also Kensinger and Corkin [446] for related discussion). Our model predicts, therefore, that performance on all tasks that require the involvement of the PFC would suffer at times of strong emotionality. However, a complete understanding of the neurobiological basis of the curvilinear versus linear components of the Yerkes-Dodson law will require additional investigation of how stress rapidly enhances, and then suppresses, hippocampal functioning.

## 14. SUMMARY

In this synthesis, we have presented our perspective on the neurobiological basis of the stress-induced enhancement and impairment of memory. First, we have asserted that the view, developed in the 1950s, that imposed a monolithic curvilinear shape on all performance-emotion interactions led to decades of debates which inappropriately called for the repeal of the Yerkes-Dodson law. We have discussed how the original version of the Yerkes-Dodson law took into account the interaction of task difficulty with arousal level to address how strong motivation can either enhance or impair performance. We recognize, however, that one problem with the Yerkes-Dodson law is that it invokes an ill-defined distinction between “simple” versus “complex” tasks. We have suggested that identifying the involvement of the PFC in a task, which can be confirmed to some degree by neuroimaging analysis, may provide a general guideline for predicting whether performance on a task in times of strong emotionality will express a linear versus nonlinear shape.

Our neurobiological model of stress-memory interactions addresses the complex, and seemingly conflicting, findings of how stress affects hippocampal LTP, and therefore, how hippocampus-dependent memory is affected by strong emotionality. We have suggested that a rapprochement can be accomplished by examining the timing between an emotional experience and a test of hippocampal functioning, as measured by hippocampus-dependent learning or LTP induction. If the two coincide in time, then hippocampal functioning would be enhanced, but if there is a substantial delay between the stress onset and either hippocampus-dependent learning or tetanizing stimulation, then measures of hippocampal functioning (memory consolidation or LTP) would be impaired. We have substantiated this model with our finding that spatial memory was enhanced when stress and spatial learning occurred in close temporal proximity, but when there was a delay between stress and learning, memory consolidation was impaired. We have also suggested that strong emotionality changes the hippocampus from a “cognitive map” mode of memory processing to a “flashbulb memory” mode, which enables the hippocampus to store disembodied fragments of an experience which lack the depth of processing of context normally attributed to hippocampal memory encoding. Overall, our model of how the hippocampus, amygdala, and PFC are differentially affected by strong emotionality provides a framework for further advancements in our understanding of the neurobiology of traumatic memory processing.
